# Dietitians’ Knowledge, Attitudes, and Practices Regarding Food–Drug and Drug–Nutrient Interactions in Saudi Arabia: A Cross-Sectional Study

**DOI:** 10.3390/healthcare14111595

**Published:** 2026-06-05

**Authors:** Howeida Abusalih, Maha M. Alsobhi, Buthaina M. Aljehany, Rowida Khader Allily, Haya Aljadani, Eman A. Abduljawad, Manal M. S. Mansoury, Sarah A. Alasmari, Afnan H. Saaty, Dalal A. Alkhudhayri, Abeer A. Aljehani, Nada Benajiba

**Affiliations:** 1 Department of Health Sciences, College of Health and Rehabilitation Sciences, Princess Nourah Bint Abdulrahman University, P.O. Box 84428, Riyadh 11671, Saudi Arabia; hhabusalih@pnu.edu.sa; 2 Food and Nutrition Department, Human Sciences and Design Faculty, King Abdulaziz University, Jeddah 21589, Saudi Arabia; malsobhi0079@stu.kau.edu.sa (M.M.A.); baljehany@kau.edu.sa (B.M.A.); rallaily@kau.edu.sa (R.K.A.); hhaljadani@kau.edu.sa (H.A.); eabduljawad@kau.edu.sa (E.A.A.); mmmansouri@kau.edu.sa (M.M.S.M.); ahsatee@kau.edu.sa (A.H.S.); aaaaljehani1@kau.edu.sa (A.A.A.); 3 Public Health Department, College of Health Sciences, Saudi Electronic University, Riyadh 11673, Saudi Arabia; sa.alasmari@seu.edu.sa; 4 Department of Home Economic, Prince Sattam Bin Abdulaziz University, Alkharj City 11942, Saudi Arabia; d.alkhudhayri@psau.edu.sa; 5 Joint Research Unit in Nutrition and Food RDC-Nutrition AFRA/IAEA, Ibn Tofail University-CNESTEN, Kenitra 14000, Morocco

**Keywords:** food–drug interactions, drug–nutrient interactions, dietitians, knowledge, attitudes and practices, Saudi Arabia

## Abstract

**Background:** Dietitians play a critical role in preventing food–drug interactions (FDIs) and drug–nutrient interactions (DNIs); however, evidence regarding their knowledge, attitudes, and practices (KAP) in Saudi Arabia remains limited. **Objective:** The aim of this study was to assess dietitians’ KAP regarding FDIs and DNIs and examine their associations with socio-demographic and professional characteristics. **Methods:** A national cross-sectional study was conducted among 353 dietitians using a validated and modified questionnaire. Knowledge was assessed via 15 multiple-choice items (score range 0–15) and categorized as poor (0–5), moderate (6–10), or good (11–15). Attitudes were assessed using 8 Likert-scale statements (score range 8–40) and classified as negative (8–19), neutral (20–29), or positive (30–40). Practices were assessed via 6 frequency-scale items (score range 6–30) and categorized as poor (6–14), moderate (15–23), or good (24–30). Associations were analyzed using chi-square tests. **Results:** In total, 65.2% of participants demonstrated poor knowledge. Knowledge level was significantly associated with nationality (*p* = 0.011), educational qualification (*p* = 0.042), attendance at FDI/DNI training courses (*p* = 0.003), and inclusion of related topics during university education (*p* = 0.013). Higher knowledge levels were also associated with managing digestive diseases (*p* = 0.001), cardiovascular diseases (*p* = 0.020), and cancer (*p* = 0.031). Positive attitudes were reported by 77.6% of participants and were significantly associated with managing cardiovascular disease (*p* < 0.001) and obesity (*p* = 0.008). Good practices were observed in 36.3% of dietitians and were significantly associated with younger age (*p* = 0.024), more recent graduation (*p* = 0.006), fewer years of professional experience (*p* = 0.002), and managing obesity (*p* = 0.016). Knowledge was positively associated with practice (*p* < 0.001). **Conclusions:** Despite generally positive attitudes, substantial gaps in knowledge and practice regarding FDIs and DNIs exist among dietitians in Saudi Arabia. Strengthening academic curricula and continuing professional education is essential to enhance competency and improve patient safety.

## 1. Introduction

Food intake and medication use are fundamental components of disease prevention and clinical management, yet their concurrent use may give rise to clinically significant food–drug interactions (FDIs) and drug–nutrient interactions (DNIs). These interactions occur when foods, nutrients, or dietary patterns alter the pharmacological effects of medications, or when medications interfere with the absorption, metabolism, or utilization of nutrients [1,2,3]. With the increasing prevalence of chronic diseases and long-term pharmacotherapy, FDIs and DNIs have emerged as important determinants of therapeutic effectiveness, patient safety, and nutritional status.

Food–drug and drug–nutrient interactions may influence both pharmacokinetic and pharmacodynamic processes. Pharmacokinetic interactions affect drug absorption, distribution, metabolism, and excretion, while pharmacodynamic interactions modify the physiological or biochemical response to a drug at its site of action [2,4]. According to the U.S. Food and Drug Administration, FDIs occur when food components or nutrients alter these processes, potentially leading to reduced therapeutic efficacy or increased risk of adverse effects [5]. Similarly, DNIs occur when medications impair nutrient absorption or metabolism, contributing to nutrient deficiencies or metabolic imbalances [6].

Several FDIs and DNIs of clinical relevance are well established in the literature. The consumption of tyramine-rich foods alongside monoamine oxidase inhibitors can precipitate hypertensive crises, while high vitamin K intake may reduce the anticoagulant efficacy of warfarin [7,8]. The absorption of tetracyclines and fluoroquinolones may be significantly reduced when taken with calcium-rich foods due to chelation effects, whereas grapefruit juice can inhibit cytochrome P450 enzymes, increasing plasma concentrations of certain medications such as statins and calcium channel blockers [9,10]. From a nutritional perspective, prolonged use of some medications, including diuretics, metformin, and proton pump inhibitors, has been associated with deficiencies in potassium, vitamin B12, magnesium, and other micronutrients [1].

The clinical implications of FDIs and DNIs extend beyond pharmacotherapy to encompass broader public health concerns. Medication-related problems, including inappropriate food–drug combinations, contribute to preventable adverse events, hospitalizations, and increased healthcare costs worldwide [2]. Effective prevention and management of these interactions, therefore, require coordinated efforts among healthcare professionals, particularly those with expertise in nutrition. A cross-comparison of existing evidence from international, Gulf, and broader Middle Eastern healthcare settings reveals a consistent pattern of FDI and DNI knowledge gaps across healthcare disciplines, shaped by regional educational and systemic factors. In Jordan, cross-sectional studies among pharmacists reported FDI knowledge scores of approximately 60–62% [5]. Among Palestinian pharmacists specifically, Altamimi et al. reported that only 53.5% demonstrated good knowledge of dietary supplement–drug interactions, with significant knowledge gaps identified despite nearly universal recognition of the clinical importance of such interactions [11]. In Yemen, a study among pharmacy college students found that the majority exhibited poor knowledge of drug–food interactions, with mean scores significantly below acceptable competency thresholds, highlighting critical deficiencies in undergraduate pharmacology–nutrition integration [12]. In Qatar, nurses achieved a mean FDI knowledge score of only 20 out of 34, with structured FDI training identified as universally absent from nursing education [13]. Collectively, these findings indicate that while attitudes toward the clinical importance of DNIs remain high (>85%), knowledge deficits and suboptimal practices persist across professional groups and geographic settings.

Dietitians play a pivotal role in identifying, preventing, and managing FDIs and DNIs through dietary assessment, patient education, and interdisciplinary collaboration. Their knowledge, attitudes, and practices are essential to ensuring safe medication use while maintaining optimal nutritional status [14]. However, evidence from multiple settings indicates that knowledge of FDIs and DNIs remains suboptimal among healthcare professionals, including physicians, nurses, pharmacists, and dietitians, despite generally positive attitudes toward their clinical importance [1,2,15].

In Saudi Arabia, available studies have reported limited awareness and knowledge of food–drug and drug–nutrient interactions among healthcare providers, with gaps attributed to insufficient training and limited integration of nutrition–pharmacology content in professional education [10,16]. Research conducted among the general population has similarly shown low awareness of FDIs, underscoring the need for competent counseling by nutrition professionals [6]. Despite this growing body of evidence, no published study has comprehensively examined the knowledge, attitudes, and practices of dietitians regarding FDIs and DNIs within the Saudi healthcare system.

Addressing this gap is particularly important given the increased recognition of dietitians’ roles in clinical and public health settings in Saudi Arabia [17]. Therefore, the present study aims to assess the knowledge, attitudes, and practices of dietitians in Saudi Arabia regarding FDIs and DNIs and to examine their associations with professional characteristics, including educational level, years of experience, and training exposure. In this study, a dietitian is defined as a healthcare professional holding at least a bachelor’s degree in clinical nutrition, licensed by the Saudi Commission for Health Specialties, and actively engaged in patient care.

## 2. Materials and Methods

### 2.1. Study Design and Subjects

A national cross-sectional study was conducted between January and November 2024 across the five administrative regions of the Kingdom of Saudi Arabia. Ethical approval was obtained from the Human Research Ethics Committee of the Faculty of Medicine, King Abdulaziz University, Jeddah, Saudi Arabia (Approval number 132-24, 2 May 2024), as well as from the Ministry of Health (Approval No. A01935, 27 May 2024).

The study population comprised licensed dietitians practicing in Saudi Arabia. Eligible participants included both male and female dietitians holding a valid professional license issued by the Saudi Commission for Health Specialties (SCFHS) and actively engaged in patient care at the time of data collection. Dietitians employed in public or private healthcare institutions, academic settings, private clinics, online nutrition services, or working as self-employed practitioners were eligible for inclusion, provided their professional practice involved clinical nutrition or dietetics. Dietitians who were not directly involved in patient care or who did not hold an active SCFHS license were excluded from the study.

Prior to participation, all eligible dietitians were provided with detailed information regarding the study objectives, procedures, and confidentiality measures. Participants were presented with an institutional review board (IRB)-approved information page and were required to actively click an “I agree” button to manifest their electronic informed consent before the questionnaire sections would unlock. Participation was entirely voluntary, and participants were informed of their right to withdraw from the study at any time without any consequences. The study posed no physical or psychological risks, and no financial or other incentives were offered. Anonymity was ensured by collecting data without personal identifiers, and all information was treated with strict confidentiality and used solely for research purposes.

### 2.2. Study Population and Sample Size

The sample size was calculated based on data reported by the Saudi Commission for Health Specialties in 2018, which estimated the total number of registered dietitians in the Kingdom to be 4144 [18]. The required sample size was calculated using the formula: N = (z^2^pq)/(d^2^). Assuming a prevalence of 50% for good practices, a 95% confidence level (z = 1.96), and a margin of error of 5%, the minimum required sample size was estimated at 352 dietitians.

### 2.3. Data Collection Procedure and Tool

A previously created and validated questionnaire by Sultan et al. (2021) was modified for use in this investigation [6]. The modifications involved the removal of five items from the knowledge section due to their irrelevance to the research aims and study population. The response style was altered from multiple-choice to single-answer questions to enhance measurement precision and minimize variability. The three-point Likert scale was modified to a five-point Likert scale in the attitudes section to enhance accuracy, improve data reliability, and mitigate answer bias by incorporating a neutral choice. In the practical section, two open-ended questions were converted into closed-ended questions to reduce attrition, improve statistical analysis, and produce more accurate findings. All adjustments were deliberated and validated with experts from nutrition and pharmacy, who provided their insights and endorsement. No translation or back-translation was required for this study. The questionnaire was developed and administered in English, as clinical nutrition education in Saudi Arabia is conducted entirely in English at both undergraduate and postgraduate levels. All participants were licensed dietitians with formal academic training in clinical nutrition, meaning the scientific and technical terminology used in the questionnaire is consistent with the professional language used in their daily practice and academic formation. The questionnaire was created via Google Forms and sent to dietitians through smartphone applications like WhatsApp, Telegram, email, Twitter, and LinkedIn. Participants were recruited using a convenience sampling approach through professional and academic networks via WhatsApp, Telegram, email, Twitter/X, and LinkedIn platforms. Eligibility criteria were clearly stated at the beginning of the online questionnaire, and only self-identified dietitians currently practicing in Saudi Arabia were eligible to participate. No independent verification of professional licensure or registration status was conducted. Although no independent verification of professional licensure or active registration was conducted, similar methodologies relying on self-reported professional status have been widely used in previous online cross-sectional studies involving healthcare professionals and dietitians [5,15,19].

The questionnaire, available in the Supplementary Materials**,** is composed of four main sections: general information, knowledge, attitudes, and practices related to FDIs and DNIs.

1. The **general information section** collected data on participants’ socio-demographic and professional characteristics. Socio-demographic variables included age, gender, year of graduation, specialty, and region or city of practice. Professional characteristics comprised years of experience, highest academic degree attained, professional qualifications, current position, healthcare sector (public or private), subspecialty (if applicable), and professional recognition or certification obtained from the Saudi Food and Drug Authority (SFDA), such as Registered Dietitian or Consultant status. In addition, information related to participants’ nutritional background was gathered, including exposure to undergraduate nutrition courses and postgraduate courses or training sessions in nutrition. This information provided insight into the overall nutrition-related educational background of the respondents.
2. The **knowledge section** assessed dietitians’ understanding of common and clinically relevant FDIs and DNIs. It consisted of multiple-choice questions addressing interactions between specific foods, nutrients, and medications, including, for example, the effect of vitamin K-rich foods on warfarin therapy, grapefruit juice interactions with cardiovascular drugs, potassium-rich foods and angiotensin-converting enzyme inhibitors, and the impact of acid-suppressing or enzyme-inducing medications on vitamin and mineral absorption. An “I don’t know” option was included for each item to reduce the likelihood of random guessing. Each correct response was awarded one point, whereas incorrect or “I don’t know” responses received zero points. The total knowledge score ranged from 0 to 15 and was categorized into tertiles (33.3%) as follows: poor knowledge (0–5), moderate knowledge (6–10), and good knowledge (11–15).
3. The **attitude section** evaluated dietitians’ perceptions and beliefs regarding FDIs and DNIs using eight statements. Responses were measured on a five-point Likert scale ranging from strongly disagree (scored as 1) to strongly agree (scored as 5). This section explored attitudes toward the importance of FDIs and DNIs, their potential severity, distinctions between FDIs and DNIs, the need for greater emphasis during undergraduate education, the importance of continuous professional development, and professional responsibility for patient counseling and pharmacovigilance reporting. The total attitude score ranged from 8 to 40 and was classified into tertiles (33.3%) as negative attitude (8–19), neutral attitude (20–29), and positive attitude (30–40).
4. The **practice section** assessed self-reported behaviors related to the identification, management, and prevention of FDIs and DNIs in clinical practice. This section included six statements evaluating the frequency with which dietitians inquire about patients’ medication use, dietary supplements, and herbal products; provide counseling on potential FDIs and DNIs; consult drug information centers or software tools; refer, document, or report interaction cases; and engage in continuing education activities to update their knowledge. Responses were recorded using a five-point frequency scale ranging from never (scored as 1) to always (scored as 5). The total practice score ranged from 6 to 30 and was categorized into tertiles (33.3%) as poor practice (6–14), moderate practice (15–23), and good practice (24–30).

For all three domains, score classifications were derived using an equal-range division of the total attainable scores into tertiles, an approach frequently adopted in KAP research when validated or universally accepted cut-off values are unavailable. Similar distribution-based methods have been applied in prior studies assessing knowledge, attitudes, and practices regarding health-related topics [20,21].

### 2.4. Content Validity Testing

Content validity was established through evaluation by a multidisciplinary panel of nine experts in nutrition and pharmacy, which is a recommended approach for ensuring adequate domain representation of questionnaire items [22,23]. Each item was independently assessed for relevance, clarity, and appropriateness using a five-point rating scale. The Content Validity Index (CVI) demonstrated excellent validity, with item-level CVI values of 1.00 for 28 items and 0.80 for one item, exceeding the minimum acceptable threshold of 0.78 for expert panels of this size [22]. At the scale level, CVI values were 99.9% using the average method and 96.6% using the universal agreement method, indicating a high level of overall content validity.

### 2.5. Pilot Test and Internal Consistency Validity

Prior to full deployment, pilot testing was conducted with a sample of 22 practicing dietitians in Saudi Arabia; upon reviewing the minor comments received from participants, appropriate modifications and adjustments were made to the survey items. The reliability of the finalized questionnaire was then verified using Cronbach’s alpha coefficients derived from this pilot cohort. Internal consistency reliability was evaluated for each questionnaire domain as well as for the overall instrument, consistent with standard psychometric recommendations [24]. Cronbach’s alpha values ranged from 0.75 to 0.85, reflecting good to excellent internal consistency [25]. Specifically, alpha coefficients were 0.81 for the knowledge section, 0.85 for attitudes, and 0.79 for practices, while the overall questionnaire achieved an alpha value of 0.75, exceeding the commonly accepted minimum reliability threshold of 0.70 for research instruments [26].

### 2.6. Data Analysis

Data were analyzed using R software version 4.1.2 (R Foundation for Statistical Computing, Vienna, Austria). Descriptive statistics were used to summarize the data with frequencies and percentages. Associations between categorical variables were initially assessed using the Chi-square (χ^2^) test. Expected cell counts were examined to verify chi-square assumptions. For variables with sparse cell frequencies (expected counts < 5), categories were collapsed where appropriate, and Fisher’s exact test was used. The inferential analyses conducted in this study should be interpreted as exploratory, as multiple bivariate comparisons were performed to examine associations between KAP outcomes and participant characteristics. Statistical significance was set at *p* < 0.05.

## 3. Results

### 3.1. Socio-Demographic Characteristics of the Participants

Table 1 presents the socio-demographic characteristics of the participants (n = 353). Most respondents were female dietitians (83.57%). Participants aged 23–29 years represented the largest age group (57.22%). The majority were Saudi nationals (92.07%). Regarding geographic distribution, 37.7% resided in the Western region, followed by the Central region (26.9%). More than half of the participants were employed in the government sector (57.22%).

In terms of educational attainment, most participants held a bachelor’s degree (80.45%), while 15.58% held a master’s degree. The majority graduated from Saudi universities (87.82%). With respect to graduation year, 60.06% graduated within the past five years, and 24.36% graduated 5–10 years ago. Regarding work experience, 69.41% reported 0–4 years of experience, and 17.00% reported 5–9 years.

The conditions most commonly managed by dietitians included obesity (74.5%), endocrine disorders such as diabetes and thyroid disease (73.4%), cardiovascular diseases (67.1%), gastrointestinal diseases (66.0%), kidney disease (60.9%), and food allergies (60.6%). In addition, 71.10% of participants reported that their college education included topics on food–drug and nutrient–drug interactions, and 41.08% reported attending related training courses.

### 3.2. Knowledge About Food–Drug and Drug–Nutrient Interactions

Table 2 summarizes the associations between sociodemographic characteristics and knowledge of food–drug and drug–nutrient interactions. The majority of participants demonstrated poor knowledge of FDIs and DNIs (n = 230, 65.2%), while 78 participants (22.1%) demonstrated moderate knowledge, and only 45 participants (12.7%) demonstrated good knowledge. Knowledge was significantly associated with nationality (*p* = 0.011), educational qualification (*p* = 0.042), attendance at training courses (*p* = 0.003), inclusion of related topics in university studies (*p* = 0.013), and management of certain diseases, including digestive diseases (*p* = 0.001), cardiovascular diseases (*p* = 0.020), and cancer (*p* = 0.031). Among participants with good knowledge, 53% attended training courses, and 80% had prior university exposure to relevant topics. Saudi dietitians represented 87% of those with good knowledge, and bachelor’s degree holders accounted for 67%. Regarding diseases, 78% of those managing digestive diseases, 80% managing cardiovascular diseases, and 29% managing cancer demonstrated good knowledge. Most other characteristics, including gender, age, geographic region, graduation year, university, workplace sector, and years of experience, were not significantly associated with knowledge (*p* > 0.05).

### 3.3. Attitudes Toward Food–Drug and Drug–Nutrient Interactions

Table 3 summarizes the associations between sociodemographic characteristics and attitudes toward food–drug and drug–nutrient interactions. The distribution of attitude was markedly more favorable, with the majority of participants reporting positive attitudes (n = 274, 77.6%). A neutral attitude was reported by 61 participants (17.3%), while only 18 participants (5.1%) expressed negative attitudes toward the clinical importance of FDIs and DNIs. Attitudes were significantly associated with managing certain diseases, including cardiovascular disease (*p* < 0.001) and obesity (*p* = 0.008). In contrast, most other characteristics—such as gender, age, nationality, geographic region, educational qualification, year of graduation, university, job sector, and course attendance—showed no significant associations (*p* > 0.05).

### 3.4. Practice Regarding Food–Drug and Drug–Nutrient Interaction

Table 4 presents the associations between sociodemographic characteristics and practice regarding food–drug and drug–nutrient interactions. The largest group reported moderate practice behaviors (n = 162, 45.9%), while good practice was reported by 128 participants (36.3%) and poor practice by 63 participants (17.8%). Significant associations were observed with age (*p* = 0.024), graduation year (*p* = 0.006), years of experience as a dietitian (*p* = 0.002), and managing obesity (*p* = 0.016). For age, 64% of participants aged 23–29 years demonstrated good practice, compared with 29% of those aged 30–39 years. Participants who graduated within the last five years accounted for 68% of those with good practice, whereas only 12% of those who graduated more than 10 years ago had good practice. Regarding years of experience, 77% of dietitians with 0–4 years of experience demonstrated good practice, compared with 14% of those with 5–9 years of experience. Among dietitians managing obesity, 77% showed moderate or good practice, compared with 60% of those with poor practice. Most other characteristics, including gender, nationality, educational qualification, workplace sector, and attendance at courses, were not significantly associated with practice (*p* > 0.05).

### 3.5. Association Between Knowledge–Practice, Knowledge–Attitude, and Attitude–Practice Scores

Table 5 illustrates the association among knowledge–practice, knowledge–attitude, and attitude–practice scores. The knowledge score was substantially positively correlated with the practice scores (r = 20.368, *p* < 0.001). However, there is no statistically significant relationship between knowledge and attitudes (r = 6.166, *p* = 0.187), nor between attitudes and practice (r = 5.908, *p* = 0.206).

Table 5 illustrates the categorical associations among the knowledge, attitude, and practice (KAP) domains. The Chi-square test revealed a highly significant association between dietitians’ baseline knowledge and their self-reported clinical practices (*p* < 0.001). Specifically, a higher proportion of participants with good knowledge demonstrated good clinical practices (55.56%) compared to those with poor knowledge (28.26%). In contrast, no statistically significant associations were observed between the knowledge and attitude domains (*p* = 0.187), nor between the attitude and practice domains (*p* = 0.206).

## 4. Discussion

This study examined the knowledge, attitudes, and practices of dietitians in Saudi Arabia regarding FDIs and DNIs. The sociodemographic and professional profile of participants revealed a workforce dominated by young, female dietitians with bachelor’s degrees and limited years of experience, a pattern that mirrors trends reported in the dietetics and clinical nutrition workforce in Saudi Arabia [19]. The predominance of dietitians managing chronic conditions such as obesity and cardiovascular disease further underscores the clinical relevance of understanding FDIs and DNIs in routine practice, as these conditions are highly prevalent in Saudi Arabia [27] and frequently require long-term pharmacotherapy with well-documented food interaction potential [28,29].

The findings show that while some dietitians demonstrated good knowledge of FDIs and DNIs, a substantial proportion exhibited poor to moderate knowledge levels. This pattern is consistent with previous studies among dietitians and other healthcare professionals in Saudi Arabia and internationally, which have consistently reported gaps in clinically relevant FDI knowledge [1,6,16,30]. Similar deficiencies have been observed across multiple healthcare disciplines in Jordan, Palestine, India, South Africa, and the United States, suggesting that FDIs and DNIs remain underemphasized in health professional education and practice [2,5].

In this study, knowledge was significantly associated with nationality, educational qualification, exposure to FDI- and DNI-related coursework, and participation in professional training programs. These findings align with earlier evidence highlighting the central role of formal education and continuing professional development in improving competence in managing FDIs and DNIs [14,15]. Dietitians involved in the management of digestive, cardiovascular, and cancer-related conditions demonstrated higher knowledge levels, likely reflecting greater exposure to medications with well-documented dietary interactions, such as anticoagulants and chemotherapeutic agents [7]. Frequent management of patients receiving complex therapeutic regimens may reinforce practical knowledge through repeated clinical exposure, an observation also reported in studies involving clinicians working in chronic disease care settings [9,10].

Notably, years of professional experience, age, and gender were not significantly associated with knowledge levels, reinforcing findings from prior studies that experiential learning alone is insufficient without structured education and regular updates on evolving drug–nutrition evidence [2,15]. This may reflect the rapidly evolving nature of clinical nutrition and pharmacotherapy, where reliance on previously acquired knowledge without continuous professional updating may reduce competency over time. Similar observations have been reported among healthcare professionals in medication safety research, where continuing education may contribute to improved clinical knowledge [31]. Overall, these results underscore the need for standardized and mandatory training on FDIs and DNIs within undergraduate curricula and continuing professional development frameworks for dietitians.

Overall, dietitians in this study demonstrated predominantly positive attitudes toward FDIs and DNIs, reflecting strong recognition of their clinical importance. This finding is consistent with studies conducted in Jordan, Malaysia, and Ghana, where healthcare professionals generally acknowledged the relevance of FDIs despite limited factual knowledge [5,14,15]. Positive attitudes were significantly associated with managing patients with cardiovascular diseases and obesity. These conditions are highly prevalent among adult Saudis [27] and are often treated with medications highly sensitive to dietary factors, such as statins, antihypertensives, and antidiabetic agents, which may explain heightened awareness and concern among dietitians managing these populations [9,10]. Healthcare professionals working with patients affected by chronic diseases are often more aware of treatment-related complications because of the long-term and multidisciplinary nature of care, which may strengthen perceptions regarding the importance of FDIs and DNIs [9]. The absence of significant associations between attitudes and demographic or educational variables suggests that general awareness of FDIs and DNIs may be widespread, even when detailed knowledge is lacking. This finding is comparable to previous KAP studies showing that positive professional attitudes toward medication safety may exist despite moderate levels of factual knowledge, possibly because patient safety principles are strongly emphasized across healthcare disciplines [5,15].

Practices related to food–drug interactions (FDIs) and drug–nutrient interactions (DNIs) were predominantly moderate, with only a limited proportion of dietitians reporting good practice behaviors. Better practices were significantly associated with younger age, more recent graduation, fewer years of professional experience, and involvement in obesity management. These findings suggest that recently trained dietitians may benefit from more updated academic curricula that increasingly emphasize evidence-based nutrition therapy, patient safety, and medication–nutrition interactions [6,15]. Similar trends have been reported in studies among dietitians, pharmacists, and other healthcare professionals, where recent graduates demonstrated greater adherence to clinical guidelines and safer medication-related practices compared with their more experienced counterparts [2,10]. The better practice patterns observed among dietitians involved in obesity management likely reflect the complexity of obesity care, which frequently involves polypharmacy and intensive dietary counseling, increasing the clinical relevance of FDIs and DNIs awareness in routine practice [7,9]. Obesity management often requires long-term monitoring and coordination with multidisciplinary teams, which may further increase opportunities for dietitians to identify and manage potential medication–nutrition interactions in clinical practice [32]. Conversely, the lack of association between practices and formal training courses may indicate variability in course quality, duration, or practical applicability, as previously reported in other healthcare settings [2,15]. Rees et al. (2020) found that interventions using mixed pedagogies, experiential learning, social learning, and organizational support mechanisms produced more positive outcomes [33]. Collectively, these findings highlight the positive impact of contemporary education and targeted clinical exposure on FDI- and DNI-related practices, while underscoring the need to extend such training opportunities to dietitians across all career stages.

Finally, the association analysis indicated a significant positive relationship between knowledge and practice, but not between knowledge and attitude or between attitude and practice. This pattern suggests that while dietitians may hold positive attitudes toward food–drug interaction management, effective practice is more directly related to actual knowledge than to attitudes alone. This aligns with evidence from the broader literature that knowledge is a critical foundation for clinical behavior change, and attitudes, while important, do not always translate into improved practice without sufficient understanding [34]. The lack of association between attitudes and practice further emphasizes the need for competency-based education that goes beyond awareness and focuses on applied clinical decision-making. Integrating case-based learning, interprofessional education, and real-world scenarios into dietetics training may help bridge this gap [14].

### Implications for Practice and Policy

These findings have important and interconnected implications for clinical practice, nutrition education, and healthcare policy in Saudi Arabia. Given dietitians’ central role in patient education and nutrition therapy, enhancing their competence in FDIs and DNIs is essential for improving medication safety and therapeutic outcomes. The significant association between university-level FDI exposure and higher knowledge indicates that standardized FDI and DNI content should be strengthened within undergraduate curricula, licensure requirements, and continuing professional development programs. Importantly, the better practice observed among younger dietitians compared with more experienced colleagues suggests that professional development program initiatives should particularly target senior clinicians trained before FDI-related content was integrated into curricula. The disconnect between positive attitudes and good practice further indicates that individual training alone is insufficient, highlighting the need for institutional safeguards such as standardized dietitian-led FDI screening protocols integrated into routine nutritional assessment. In addition, the positive association between knowledge and practice suggests that better-informed dietitians practice more safely; however, no single profession can fully address the complexity of FDIs and DNIs. Therefore, structured interdisciplinary collaboration between dietitians, pharmacists, and physicians should be encouraged, particularly through formalized dietitian–pharmacist medication review processes for patients receiving complex long-term pharmacotherapy. Moreover, interprofessional education initiatives involving nutrition, pharmacy, and medical students through shared FDI case-based learning may better prepare future healthcare professionals for collaborative clinical practice [35].

## 5. Limitations

This investigation is the first study conducted in Saudi Arabia to assess dietitians’ knowledge, attitudes, and practices regarding FDIs and DNIs, and to examine their associations with professional and demographic characteristics. Data were collected using a modified questionnaire that demonstrated acceptable validity and reliability, enhancing its suitability for the target population and its ability to capture the study objectives. Nevertheless, several limitations should be considered when interpreting the findings. First, the cross-sectional design precludes the establishment of causal relationships between knowledge, attitudes, practices, and their associated factors [36,37]. Second, the convenience sampling approach [38], and online distribution of the questionnaire through professional digital platforms may have disproportionately reached dietitians who are more digitally engaged and professionally networked, potentially overestimating FDI and DNI knowledge and practice levels relative to the broader dietetics workforce. Consequently, although participants were recruited from multiple regions of Saudi Arabia, the absence of probability-based sampling means that the term “national” should be interpreted cautiously, as the findings may not be fully representative of all practicing dietitians in the Kingdom [39,40]. Related to this, the online survey format precludes the calculation of a precise response rate, as the total number of dietitians who received and viewed the invitation cannot be determined, introducing a further source of non-response bias [40]. Third, the reliance on self-reported data for the attitude and practice sections may have introduced social desirability bias, whereby participants overreported favorable attitudes or positive clinical behaviors [41]. To mitigate guessing bias in the knowledge section, an “I don’t know” response option was included, consistent with recommendations for healthcare KAP surveys [42]. Fourth, the analyses relied on bivariate statistical tests and did not include multivariable regression models to control for potential confounders or interrelationships among variables such as age, years of experience, and graduation year. Future studies employing ordinal or multinomial logistic regression are recommended to identify independent predictors of knowledge, attitudes, and practices while adjusting for covariates. Fifth, while construct validity was not formally assessed through confirmatory factor analysis—given that the instrument was adapted from a previously validated tool rather than newly developed—the adapted questionnaire demonstrated acceptable content validity and internal consistency reliability, as described in the Methods section. Future research employing longitudinal or interventional designs is recommended to evaluate the impact of structured educational and training programs on improving dietitians’ FDI and DNI competencies, and registry-based random sampling stratified by region and professional setting would substantially strengthen the representativeness and generalizability of findings.

## 6. Conclusions

While dietitians in Saudi Arabia demonstrate generally positive attitudes toward FDIs and DNIs, gaps in knowledge and inconsistencies in practice remain evident. Knowledge was associated with practice, underscoring the importance of structured education and continuous professional training. Addressing these gaps is crucial to enhancing patient safety and optimizing therapeutic outcomes in an increasingly complex healthcare environment.

## Figures and Tables

**Table 1:** Socio-demographic characteristics of the studied population (N = 353) (n, %).

Variables	N	%
Gender		
Male	58	16.43
Female	295	83.57
Age (years)		
23–29 years	202	57.22
30–39 years	126	35.69
≥40 years	25	7.27
Nationality		
Saudi	325	92.07
Non-Saudi	28	7.93
Geographical area		
Western	133	37.68
Eastern	51	14.45
Southern	35	9.92
Central	95	26.91
Northern	39	11.05
Educational Qualification		
Bachelor’s	284	80.45
Master’s	55	15.58
Doctorate	14	3.97
Graduation Year		
Before 5 years ago	212	60.06
5–10 years ago	86	24.36
More than 10 years ago	55	15.58
Graduation university		
Saudi Arabia	310	87.82
Non-Saudi	43	12.18
Type of Workplace sector		
Government	202	57.22
Private	151	42.78
Years of Experience as a dietitian		
0–4	245	69.41
5–9	60	17.00
10–14	35	9.92
≥15	13	3.68
Have you attended courses on food–drug interactions and nutrient–drug interaction?		
Yes	145	41.08
No	156	44.19
I don’t remember	52	14.73
Did your university studies include subjects related to food–drug interactions and nutrient–drug interaction?		
Yes	251	71.10
No	65	18.41
I don’t remember	37	10.48
What diseases do you deal with or have dealt with in your practice as a dietitian?		
Digestive Diseases such as Peptic Ulcer, Ulcerative Colitis, IBS, Celiac disease	233	66.01
Endocrine Diseases (Diabetes, thyroid)	259	73.37
Cardiovascular Diseases (Hypertension, etc.)	237	67.14
Inherited Metabolic Disorders	85	24.08
Food Allergies	214	60.62
Kidney Disease	215	60.91
Mental Health	61	17.28
Obesity	263	74.50
Cancer	107	30.31

**Table 2:** Association between sociodemographic characteristics and knowledge regarding food–drug/drug–nutrient interactions.

Variables	Knowledge N, %			χ^2^	*p*-Value
	Poor	Moderate	Good		
Gender				1.204	0.548
Male	39 (16.96%)	10 (12.82%)	9 (20.0%)		
Female	191 (83.04%)	68 (87.18%)	36 (80.0%)		
Age (years)				Fisher’s exact test	0.26
23–29 years	135 (58.70%)	48 (61.54%)	19 (42.22%)		
30–39 years	80 (34.78%)	24 (30.77%)	22 (48.89%)		
≥40 years	15 (6.52%)	6 (7.69%)	4 (8.89%)		
Nationality				8.988	0.011 *
Saudi	219 (95.22%)	67 (85.90%)	39 (86.67%)		
Non-Saudi	11 (4.78%)	11 (14.10%)	6 (13.33%)		
Geographical area				6.332	0.610
Western	83 (36.09%)	33 (42.31%)	17 (37.78%)		
Eastern	32 (13.91%)	12 (15.38%)	7 (15.56%)		
Southern	22 (9.57%)	9 (11.54%)	4 (8.89%)		
Central	61 (26.52%)	20 (25.64%)	14 (31.11%)		
Northern	32 (13.91%)	4 (5.13%)	3 (6.67%)		
Educational Qualification				9.923	0.042 *
Bachelor’s	193 (83.91%)	61 (78.21%)	30 (66.66%)		
Master’s	32 (13.91%)	12 (15.38%)	11 (24.44%)		
Doctorate	5 (2.17%)	5 (6.41%)	4 (8.89%)		
Graduation Year				4.628	0.328
Before 5 years ago	132 (57.39%)	55 (70.51%)	25 (55.56%)		
5–10 years ago	60 (26.09%)	14 (17.95%)	12 (26.67%)		
More than 10 years ago	38 (16.52%)	9 (11.54%)	8 (17.78%)		
Graduation university				4.376	0.112
Saudi Arabia	208 (90.43%)	64 (82.05%)	38 (84.44%)		
Non-Saudi	22 (9.57%)	14 (17.95%)	7 (15.56%)		
Type of Workplace sector				2.350	0.309
Government	135 (58.70%)	46 (58.97%)	21 (46.67%)		
Private	95 (41.30%)	32 (41.03%)	24 (53.33%)		
Years of Experience as a dietitian				Fisher’s exact test	0.79
0–4	163 (70.87%)	53 (67.95%)	29 (64.44%)		
5–9	34 (14.78%)	16 (20.51%)	10 (22.22%)		
10–14	23 (10.00%)	7 (8.97%)	5 (11.11%)		
≥15	10 (4.34%)	2 (2.56%)	1 (2.22%)		
Have you attended courses on food–drug interactions and nutrient–drug interaction?				15.745	0.003 *
Yes	80 (34.78%)	41 (52.56%)	24 (53.33%)		
No	112 (48.70%)	32 (41.03%)	12 (26.67%)		
I don’t remember	38 (16.52%)	5 (6.41%)	9 (20.00%)		
Did your university studies include subjects related to food–drug interactions and nutrient–drug interaction?				12.573	0.013 *
Yes	150 (65.22%)	65 (83.33%)	36 (80.00%)		
No	53 (23.04%)	8 (10.26%)	4 (8.89%)		
I don’t remember	27 (11.74%)	5 (6.41%)	5 (11.11%)		
What diseases do you deal with or have dealt with in your practice as a dietitian?					
Digestive Diseases	136 (59.13%)	62 (79.49%)	35 (77.78%)	13.943	0.001 *
Endocrine Diseases	161 (70.00%)	60 (76.92%)	38 (84.44%)	4.666	0.097
Cardiovascular Diseases	143 (62.17%)	58 (74.36%)	36 (80.00%)	7.787	0.020 *
Inherited Metabolic Disorders	53 (23.04%)	16 (20.51%)	16 (35.56%)	3.920	0.141
Food Allergies	141 (61.30%)	45 (57.69%)	28 (62.22%)	0.374	0.830
Kidney Disease	135 (58.70%)	50 (64.10%)	30 (66.67%)	1.434	0.488
Mental Health	33 (14.35%)	20 (25.64%)	8 (17.78%)	5.206	0.074
Obesity	167 (72.61%)	58 (74.36%)	38 (84.44%)	2.777	0.249
Cancer	61 (26.52%)	33 (42.31%)	13 (28.89%)	6.921	0.031 *
Total	230	78	45		

A *p*-value < 0.05 (*) indicates statistical significance.

**Table 3:** Association between sociodemographic characteristics and attitude regarding food–drug/drug–nutrient interactions.

Variables	Attitude N, %			χ^2^	*p*-Value
	Poor	Moderate	Good		
Gender				5.955	0.051
Male	5 (27.78%)	15 (24.59%)	38 (13.87%)		
Female	13 (72.22%)	46 (75.41%)	236 (86.13%)		
Age (years)				Fisher’s exact test	0.93
23–29 years	11 (61.11%)	34 (55.74%)	157 (57.30%)		
30–39 years	5 (27.78%)	22 (36.07%)	99 (36.13%)		
≥40 years	2 (11.11%)	5 (8.20%)	18 (6.57%)		
Nationality				0.410	0.814
Saudi	16 (88.89%)	57 (93.44%)	252 (91.97%)		
Non-Saudi	2 (11.11%)	4 (6.56%)	22 (8.03%)		
Geographical area				7.331	0.501
Western	5 (27.78%)	19 (31.15%)	109 (39.78%)		
Eastern	4 (22.22%)	12 (19.67%)	35 (12.77%)		
Southern	3 (16.67%)	6 (9.84%)	26 (9.49%)		
Central	6 (33.33%)	16 (26.23%)	73 (26.64%)		
Northern	0	8 (13.11%)	31 (11.31%)		
Educational Qualification				7.612	0.107
Bachelor’s	16 (88.89%)	47 (77.05%)	221 (80.66%)		
Master’s	2 (11.11%)	8 (13.11%)	45 (16.42%)		
Doctorate	0	6 (9.84%)	8 (2.92%)		
Graduation Year				4.085	0.395
Before 5 years ago	14 (77.78%)	32 (52.46%)	166 (60.58%)		
5–10 years ago	3 (16.67%)	18 (29.51%)	65 (23.72%)		
More than 10 years ago	1 (5.56%)	11 (18.03%)	43 (15.69%)		
Graduation university				0.073	0.964
Saudi Arabia	16 (88.89%)	53 (86.89%)	241 (87.96%)		
Non-Saudi	2 (11.11%)	8 (13.11%)	33 (12.04%)		
Type of Workplace sector				3.900	0.142
Government	14 (77.78%)	37 (60.66%)	151 (55.11%)		
Private	4 (22.22%)	24 (39.34%)	123 (44.89%)		
Years of Experience as a dietitian				Fisher’s exact test	0.95
0–4	12 (66.67%)	38 (62.30%)	195 (71.17%)		
5–9	3 (16.67%)	12 (19.67%)	45 (16.42%)		
10–14	2 (11.11%)	8 (13.11%)	25 (9.12%)		
≥15	1 (5.56%)	3 (4.92%)	9 (3.28%)		
Have you attended courses on food–drug interactions and nutrient–drug interaction?				7.048	0.133
Yes	12 (66.67%)	25 (40.98%)	108 (39.42%)		
No	4 (22.22%)	24 (39.34%)	128 (46.72%)		
I don’t remember	2 (11.11%)	12 (19.67%)	38 (13.87%)		
Did your university studies include subjects related to food–drug interactions and nutrient–drug interaction?				1.716	0.788
Yes	14 (77.78%)	40 (65.57%)	197 (71.90%)		
No	2 (11.11%)	13 (21.31%)	50 (18.25%)		
I don’t remember	2 (11.11%)	8 (13.11%)	27 (9.85%)		
What diseases do you deal with or have dealt with in your practice as a dietitian?					
Digestive Diseases	11 (61.11%)	33 (54.10%)	189 (68.98%)	5.126	0.077
Endocrine Diseases	11 (61.11%)	39 (63.93%)	209 (76.28%)	5.350	0.070
Cardiovascular Diseases	8 (44.44%)	30 (49.18%)	199 (72.63%)	16.861	<0.001 *
Inherited Metabolic Disorders.	6 (33.33%)	9 (14.75%)	70 (25.55%)	4.068	0.131
Food Allergies.	9 (50.00%)	35 (57.38%)	170 (62.04%)	1.352	0.509
Kidney Disease.	8 (44.44%)	33 (54.10%)	174 (63.50%)	4.012	0.134
Mental Health.	5 (27.78%)	10 (16.39%)	46 (16.79%)	1.468	0.480
Obesity.	13 (72.22%)	36 (59.02%)	214 (78.10%)	9.620	0.008 *
Cancer.	7 (38.89%)	12 (19.67%)	88 (32.12%)	4.318	0.115
Total	18	61	274		

A *p*-value < 0.05 (*) indicates statistical significance.

**Table 4:** Association between sociodemographic characteristics and practice regarding food–drug/drug–nutrient interactions.

Variables	Practice N, %			χ^2^	*p*-Value
	Poor	Moderate	Good		
Gender				0.778	0.678
Male	8 (12.70%)	28 (17.28%)	22 (17.19%)		
Female	55 (87.30%)	134 (82.72%)	106 (82.81%)		
Age (years)				Fisher’s exact test	0.01 *
23–29 years	24 (38.10%)	96 (59.26%)	82 (64.06%)		
30–39 years	35 (55.56%)	54 (33.33%)	37 (28.91%)		
≥40 years	4 (6.35%)	12 (7.41%)	9 (7.03%)		
Nationality				0.642	0.725
Saudi	59 (93.65%)	150 (92.59%)	116 (90.63%)		
Non-Saudi	4 (6.35%)	12 (7.41%)	12 (9.37%)		
Geographical area				9.104	0.334
Western	19 (30.16%)	59 (36.42%)	55 (42.97%)		
Eastern	12 (19.05%)	21 (12.96%)	18 (14.06%)		
Southern	4 (6.35%)	17 (10.49%)	14 (10.94%)		
Central	22 (34.92%)	48 (29.63%)	25 (19.53%)		
Northern	6 (9.52%)	17 (10.49%)	16 (12.50%)		
Educational Qualification				0.590	0.964
Bachelor’s	50 (79.37%)	132 (81.48%)	102 (79.69%)		
Master’s	11 (17.46%)	23 (14.20%)	21 (16.41%)		
Doctorate	2 (3.17%)	7 (4.32%)	5 (3.91%)		
Graduation Year				14.587	0.006 *
Before 5 years ago	25 (39.68%)	100 (61.73%)	87 (67.97%)		
5–10 years age	24 (38.10%)	37 (22.84%)	25 (19.53%)		
More than 10 years ago	14 (22.22%)	25 (15.43%)	16 (12.50%)		
Graduation university				3.750	0.153
Saudi Arabia	51 (80.95%)	143 (88.27%)	116 (90.63%)		
Non-Saudi	12 (19.05%)	19 (11.73%)	12 (9.38%)		
Type of Workplace sector				0.092	0.955
Government	36 (57.14%)	94 (58.02%)	72 (56.25%)		
Private	27 (42.86%)	68 (41.98%)	56 (43.75%)		
Years of Experience as a dietitian				Fisher’s exact test	0.001 *
0–4	31 (49.21%)	115 (70.99%)	99 (77.34%)		
5–9	20 (31.75%)	22 (13.58%)	18 (14.06%)		
10–14	11 (17.46%)	16 (9.88%)	8 (6.25%)		
≥15	1 (1.59%)	9 (5.55%)	3 (2.34%)		
Have you attended courses on food–drug interactions and nutrient–drug interaction?				9.309	0.054
Yes	18 (28.57%)	64 (39.51%)	63 (49.22%)		
No	35 (55.56%)	76 (46.91%)	45 (35.16%)		
I don’t remember	10 (15.87%)	22 (13.58%)	20 (15.63%)		
Did your university studies include subjects related to food–drug interactions and nutrient–drug interaction?				4.174	0.383
Yes	43 (68.25%)	112 (69.14%)	96 (75.00%)		
No	15 (23.81%)	33 (20.37%)	17 (13.28%)		
I don’t remember	5 (7.94%)	17 (10.49%)	15 (11.72%)		
What diseases do you deal with or have dealt with in your practice as a dietitian?					
Digestive Diseases	38 (60.32%)	106 (65.43%)	89 (69.53%)	1.641	0.440
Endocrine Diseases	46 (73.02%)	120 (74.07%)	93 (72.66%)	0.078	0.961
Cardiovascular Diseases	45 (71.43%)	102 (62.96%)	90 (70.31%)	2.390	0.303
Inherited Metabolic Disorders.	9 (14.29%)	40 (24.69%)	36 (28.13%)	4.485	0.106
Food Allergies.	37 (58.73%)	101 (62.35%)	76 (59.38%)	0.379	0.827
Kidney Disease.	35 (55.56%)	97 (59.88%)	83 (64.84%)	1.663	0.435
Mental Health.	10 (15.87%)	30 (18.52%)	21 (16.41%)	0.329	0.848
Obesity	38 (60.32%)	127 (78.40%)	98 (76.56%)	8.252	0.016 *
Cancer	18 (28.57%)	47 (29.01%)	42 (32.81%)	0.599	0.741
Total	63	162	128		

A *p*-value < 0.05 (*) indicates statistical significance.

**Table 5:** Association between knowledge–practice, knowledge–attitude, and attitude–practice scores.

Characteristics	Knowledge			χ^2^	*p*-Value
	Poor	Moderate	Good		
**Practice**				20.368	<0.001 *
Poor	47 (20.43%)	13 (16.67%)	3 (6.67%)		
Moderate	118 (51.30%)	27 (34.62%)	17 (37.78%)		
Good	65 (28.26%)	38 (48.72%)	25 (55.56%)		
	**Knowledge**				
	Poor	Moderate	Good		
**Attitude**				6.166	0.187
Poor	12 (5.22%)	3 (3.85%)	3 (6.67%		
Moderate	47 (20.43%)	11 (14.10%)	3 (6.67%)		
Good	171 (74.35%)	64.00 (82.05%)	39 (86.67%)		
	**Attitude**				
	Poor	Moderate	Good		
**Practice**				5.908	0.206
Poor	4 (22.22%)	11 (18.03%)	48 (17.52%)		
Moderate	9 (50.00%)	35 (57.38%)	118 (43.07%)		
Good	5 (27.78%)	15 (24.59%)	108 (39.42%)		

A *p*-value < 0.05 (*) indicates statistical significance.

## Data Availability

The original contributions presented in this study are included in the article/Supplementary Materials. Further inquiries can be directed to the corresponding author.

## Supplementary Materials

The following supporting information can be downloaded at: https://www.mdpi.com/article/10.3390/healthcare14111595/s1, File S1: Food drug/drug-nutrient interactions questionnaire for Dietitians in the Kingdom of Saudi Arabia: (FDIQ, DNIQ).
